# Successful Management of Repetitive Urinary Obstruction and Anuria Caused by Double J Stent Calculi Formation after Renal Transplantation

**DOI:** 10.1155/2014/245724

**Published:** 2014-07-06

**Authors:** Zongyao Hao, Li Zhang, Jun Zhou, Xiansheng Zhang, Haoqiang Shi, Yifei Zhang, Pengfei Wei, Chaozhao Liang

**Affiliations:** ^1^Department of Urology, The First Affiliated Hospital of Anhui Medical University and Institute of Urology, Anhui Medical University, Hefei, Anhui 230022, China; ^2^School of Life Sciences, University of Science and Technology of China, Hefei, Anhui 230027, China

## Abstract

This report firstly describes an extremely rare case of repetitive double J stent calculi formation after renal transplantation caused by the antihyperparathyroidism (HPT) drug calcitriol. In 2012, a woman initially presented to our hospital for anuria with lower abdominal pain. She was diagnosed with allograft hydronephrosis and double J stents obstruction by calculi formation after transplantation and treated with triplicate stents replacements in another hospital without clinical manifestations improvements. Through detailed exploration of medical history, we conclude that the abnormal calculi formation is due to the calcitriol (1,25-dihydroxyvitamin D3) administration, a drug which can increase renal tubular reabsorption of calcium for treating posttransplant HPT bone disease. After discontinuing calcitriol, the patient was stone-free and had a good recovery without severe complications during the 9-month follow-up. Our novel findings may provide an important clue and approach to managing formidable repetitive double J stent calculi formation in the clinical trial.

## 1. Introduction

Urinary obstruction is one of the most common urologic complications after renal transplantation, which is associated with significant morbidity and prolonged hospitalization and frequently requires other surgical interventions [1]. This complication has been gradually treated with mature techniques of retrograde ureteral stenting, which may offer an appropriate primary and definitive alternative to open surgery for its minimal invasion and considerable safety [2]. Double J stent is a soft tube designed to run from the kidney to the bladder and prevent or relieve a blockage in the ureter. We report here, for the first time, a case of ureteral obstruction and anuria after renal transplantation, in which the patient has abnormally received the repetitive double J stent interventions. After careful and detailed exploration of the etiology, we address that obstruction and anuria caused by the aberrant formation of calculi in the stent should be facilitated by the administration of calcitriol, a drug which is traditionally applied for the management of hyperparathyroidism (HPT) and osteoporosis after renal transplantation [3].

## 2. Case Report

In December 2012, a 39-year-old Chinese woman complaining of anuria with lower abdominal pain visited our hospital. She had not received unremarkable medical treatments before except for the right lateral allogeneic renal transplantation in 2009 at another hospital (hereafter referred to as Hospital A) owing to uremia. Initially, she had a good postoperative recovery via routinely taking the antirejection immunosuppressants cyclosporin A and prednisone. Simultaneously, she took calcitriol to manage commonly encountered HPT and osteoporosis after renal transplantation [3]. However, she had undergone anuria with lower abdominal pain 6 months ago, which was diagnosed as acute unilateral ureteral obstruction. Subsequently, the patient had been receiving retrograde internal 6F ureteral double J stenting provided by Bard-InLay and discharged from Hospital A. Unfortunately, she had been harassed by the identical symptoms 2 months later. Although Bard-InLay stent was the most commonly used auxiliary implement in urological interventions for its tapered tip and lubricious coating to facilitate smooth insertion and effective negotiation around obstructions, clinicians in Hospital A had substituted domestic ureteral stent brand of YZB/Jiangsu-0038-2004 for Bard-InLay, in view of different adaptabilities to the stents caused by racial diversity and personality. Surprisingly, only 3 weeks later, it is necessary for the patient to receive the third double J stent placement to solve the extremely same problem of anuria and abdominal symptoms. Even so, this last try in Hospital A was still invalid after one month.

**Figure 1:**
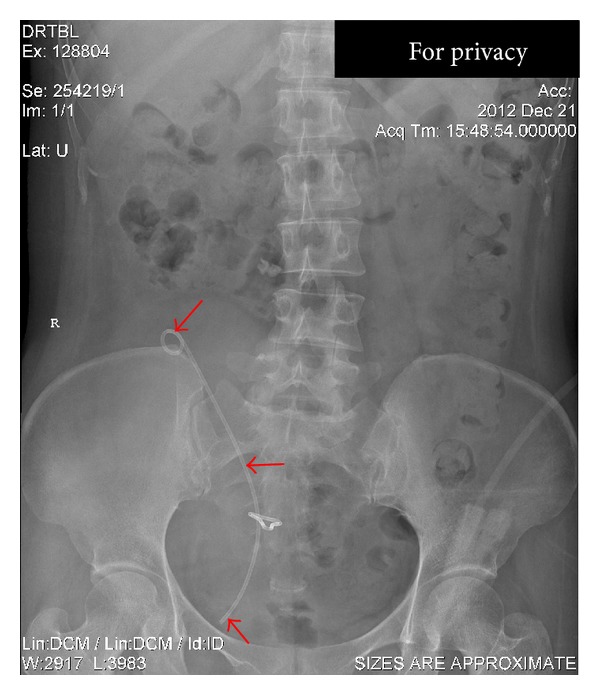
Preoperative kidney-ureter-bladder radiography image. The modified (by cutting a length of 9 cm from one end of the classical 25 cm full length) double J stent run from kidney to bladder was indicated with the red arrows.

**Figure 2:**
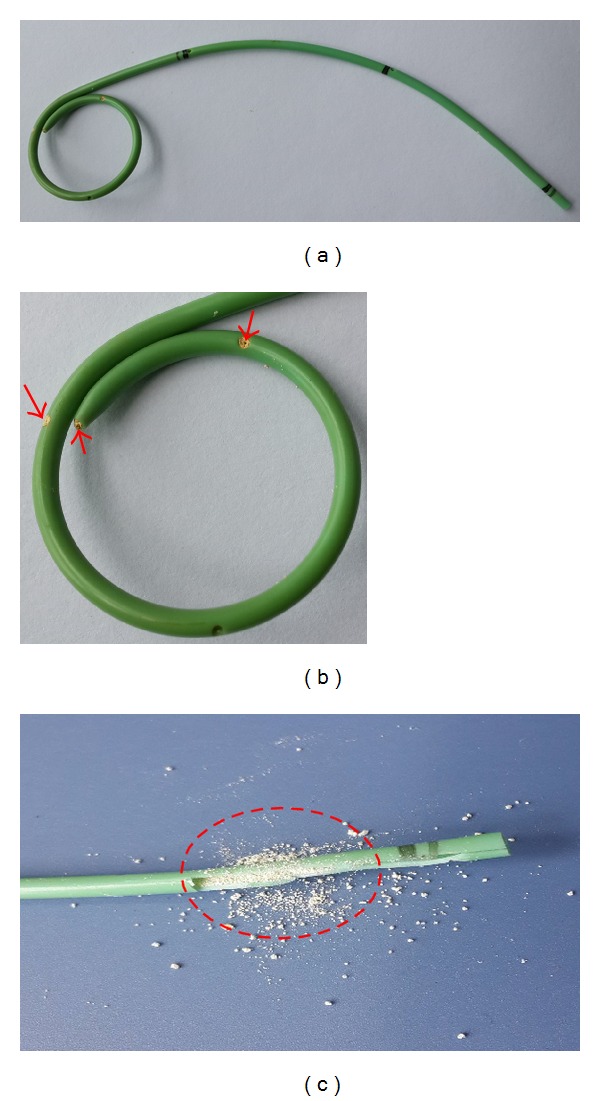
Calculi formation in the completely blocked double J stent. (a) Overview of the modified double J stent obtained from the patient's pelvic cavity. (b) Enlarged image of the double J stent: typical calculi were indicated with the red arrows. (c) Completed blockage of the double J stent by the calculi: typical calculi were indicated with the red dashed lines.

After viewing the medical history, we began to make a physical examination for her. She had unremarkable physical signs except for a suffering moon face and slight abdominal bulge and pain. Routine blood investigation file revealed red blood cells, white blood cells, and platelet counts of 4.11 × 10^12^/L, 10.38 × 10^9^/L, and 322 × 10^9^/L, respectively. Besides, the following data were obtained: total protein, 54.8 g/L; albumin, 29.0 g/L; glucose, 4.69 mmol/L; urea nitrogen, 2.84 mmol/L; creatinine, 61 *μ*mol/L; potassium, 3.30 mmol/L; sodium, 135.5 mmol/L; chlorinum, 103.1 mmol/L; magnesium, 0.63 mmol/L; phosphonium, 1.00 mmol/L; intact PTH, 32.1 pg/mL; 24-hour urine calcium, 4.42 mmol/L; and calcium, 2.19 mmol/L. For the imaging diagnosis, type-B ultrasound revealed moderate hydronephrosis of the transplanted kidney in the right lateral of pelvic cavity. Kidney-ureter-bladder (KUB) radiography clearly showed the stent modified by cutting a length of 9 cm from one end of the classical 25 cm full-length double J ureteral stent to improve its clinical acceptability (as the donor kidney is usually placed in a lower position in the pelvic cavity) (Figure 1, red arrows, and Figure 2(a)). After careful and detailed examinations, we conducted the operation to substitute a new Bard-InLay internal 6F double J stent for the originally blocked one. Subsequently, infrared spectrum analysis elucidated that the calculi in the completely blocked stent consisted of whewellite, weddellite, and carbonated apatite (Figures 2(b) and 2(c), red arrows and dashed lines). The patient's urine volume reached 4300 mL the next whole day after operation. Besides, her kidney function tests and multiple urine cytologies were absolutely normal. After discontinuing calcitriol, the patient was found to be calculi-free and the 9-month follow-up showed a good recovery without stent obstruction or anuria with lower abdominal pain, which she had been confronting several times before receiving the appropriate management in our hospital.

## 3. Discussion

Renal transplantation has been an effective and widely applied treatment for patients with end-stage renal disease. The most common complications such as urinary obstruction during the early posttransplantation period have been reported to occur at a relatively high rate. Ureteral double J stents are routinely used to prevent or relieve a blockage in the ureter [4]. Complications such as migration, fragmentation, encrustation, and even stones formation might occur in the stents, generally when they were left in place for a long time [5]. However, it was extremely rare to find repetitive stents calculi formation in a relatively short period of less than 2 months, especially even 20 days. Based upon her medical history, we speculated that the three kinds of drugs she took might be the pivotal factors to cause the stents blockage and subsequent clinical manifestations. Among them, cyclosporin A and prednisone are widely accepted to apply in the clinical prevention of allograft rejection without obvious adverse effects such as calculi formation [6]. However, calcitriol, which reversed posttransplant HPT bone disease, could increase blood calcium levels by promoting renal tubular reabsorption of calcium and reducing the loss of calcium in the urine [7]. Specifically, calcitriol is considered to be of great importance in Ca^2+^ homeostasis regulation, because it can enhance the active Ca^2+^ absorption in small intestine and promotes Ca^2+^ reabsorption in kidney [8]. Moreover, the Ca^2+^ influx protein epithelial Ca^2+^ channel (ECaC) [9], which acts as the gatekeeper and constitutes the rate-limiting step in the process of active Ca^2+^ reabsorption by promoting apical Ca^2+^ influx into mammalian distal renal tubule, has been elucidated to locate in calcitriol-responsive epithelia [10]. From the above-mentioned pharmacologic action, we supposed that calcitriol might facilitate the calculi formation, as the patient's stones were mainly composed of calcium compounds. Intriguingly, no report has shown that routine dose of calcitriol (0.25 *μ*g/day) which the patient took for approximately 3 years (ever since 2 months after renal transplantation until we set the medical order) could cause severe adverse effects to the patients when the calcium level did not exceed the normal range (2.25–2.75 mmol/L) by 0.25 mmol/L [11]. Notably, the patient's serum calcium level (2.19 mmol/L), urine calcium for 24 hours (4.42 mmol/L, reference 2.5–7.5 mmol/L), and intact PTH level (32.1 pg/mL, reference 15–65 pg/mL) were all within the normal range. Thus, we hypothesized that stents calculi formation in this case might be caused by drug-induced metabolic disorders or some unclear synergistic effects of calcitriol and some components within human body [12, 13].

## 4. Conclusion

In summary, double J stent is effective to relieve ureter blockage, generally without severe complications. However, if the complications such as calculi formation occurred, clinicians should not change the stents merely without exploring the intrinsic disorders. For the similar cases, clinicians should be aware of the patient's detailed medical history and pay great attention to the administration of the calcium-forming drugs and also should carefully monitor the patient's serum calcium level to adjust the dosage for preventing potential adverse events.
